# Planar Cell Polarity Aligns Osteoblast Division in Response to Substrate Strain

**DOI:** 10.1002/jbmr.2377

**Published:** 2015-02-16

**Authors:** Gabriel L Galea, Lee B Meakin, Dawn Savery, Hanna Taipaleenmaki, Peter Delisser, Gary S Stein, Andrew J Copp, Andre J van Wijnen, Lance E Lanyon, Joanna S Price

**Affiliations:** 1 School of Veterinary Sciences, University of BristolBristol, UK; 2 Institute of Child Health, University College LondonLondon, UK; 3 Heisenberg-Group for Molecular Skeletal Biology, Department of Trauma, Hand and Reconstructive Surgery, University Medical Center Hamburg-EppendorfHamburg, Germany; 4 University of Vermont, BurlingtonVT, USA; 5 Mayo Clinic, RochesterMN, USA

**Keywords:** OSTEOBLASTS, PLANAR CELL POLARITY, WNT SIGNALING, MECHANICAL STRAIN, VANGL2

## Abstract

Exposure of bone to dynamic strain increases the rate of division of osteoblasts and also influences the directional organization of the cellular and molecular structure of the bone tissue that they produce. Here, we report that brief exposure to dynamic substrate strain (sufficient to rapidly stimulate cell division) influences the orientation of osteoblastic cell division. The initial proliferative response to strain involves canonical Wnt signaling and can be blocked by sclerostin. However, the strain-related orientation of cell division is independently influenced through the noncanonical Wnt/planar cell polarity (PCP) pathway. Blockade of Rho-associated coiled kinase (ROCK), a component of the PCP pathway, prevents strain-related orientation of division in osteoblast-like Saos-2 cells. Heterozygous *loop-tail* mutation of the core PCP component van Gogh-like 2 (Vangl2) in mouse osteoblasts impairs the orientation of division in response to strain. Examination of bones from Vangl2 *loop-tail* heterozygous mice by µCT and scanning electron microscopy reveals altered bone architecture and disorganized bone-forming surfaces. Hence, in addition to the well-accepted role of PCP involvement in response to developmental cues during skeletal morphogenesis, our data reveal that this pathway also acts postnatally, in parallel with canonical Wnt signaling, to transduce biomechanical cues into skeletal adaptive responses. The simultaneous and independent actions of these two pathways appear to influence both the rate and orientation of osteoblast division, thus fine-tuning bone architecture to meet the structural demands of functional loading. © 2014 The Authors. *Journal of Bone and Mineral Research* published by Wiley Periodicals, Inc. on behalf of the American Society for Bone and Mineral Research.

## Introduction

The mechanisms by which the bony skeleton tunes itself to be strong enough to withstand fracture, yet not so massive as to incur disadvantageous energetic costs during locomotion,^1^,^2^ are collectively termed functional adaptation. This collection of processes involves forming, removing, and orienting bone tissue according to the increased, decreased, or directionally altered requirements for strength that arise from changing loading behavior locally in each site of the skeleton.^3^ The local strains generated by loading are presumed to act as potent environmental cues to ensure not only that there are sufficient bone-forming cells to produce sufficient new bone tissue but also to inform the prevailing orientation of the bone tissue that they produce. This mechanical influence on bone architecture is widely described as Wolff's law.^4^,^5^ Strain direction also influences bone microarchitectural properties, including the orientation of collagen fibers.^6^

The cellular mechanisms that regulate the translation of mechanical signals into appropriate bone strength remain unclear, although in recent years it has become increasingly apparent that there is no single “linear” pathway, but instead several nonspecific signaling pathways interact to produce the final effect. These pathways are likely to play distinct roles at different times, in different cells, and at different sites in the skeleton.^7^–^9^ Notwithstanding this multiplicity of contributions, there is one pathway that clearly plays a major role in adaptive bone (re)modeling and that is the canonical Wnt signaling system. Mechanical strain activates canonical Wnt/β-catenin signaling in osteoblastic cells, and inhibition of this pathway prevents the strain-related increase in osteoblastic cell proliferation.^10^–^13^ In vivo loss of β-catenin in osteocytes abrogates the adaptive response to mechanical loading,^14^ and disuse resulting in bone loss is associated with reduced β-catenin signaling.^15^ This loading-related regulation of canonical Wnt signaling involves altered expression of the osteocyte-derived antagonist sclerostin. Mechanical loading normally downregulates sclerostin expression, which is associated with an increase in canonical Wnt signaling and new bone formation.^16^–^18^ Mice lacking *Sost* can still respond to artificial mechanical loading,^19^ but they do not lose bone with disuse.^15^ Interestingly, genetically modified mice lacking sclerostin do not show grossly abnormal skeletal patterning,^20^ suggesting that the cellular processes involved in establishing and adapting the directional orientation of bone structure can be achieved without the involvement of canonical Wnt signaling.

This finding led us to explore the contribution of the noncanonical Wnt planar cell polarity (PCP) pathway in the directional realignment of osteoblast division after strain and how it may “target” cellular activity through the strategic placement of daughter osteoblasts. During development, PCP signaling substantially regulates cell directional realignment of division^21^–^24^ and plays an important role in regulating cell polarization,^25^ leading to the suggestion that it is the “missing link in skeletal morphogenesis.”^26^

PCP signaling forms a β-catenin-independent branch of Wnt signaling activated by PCP ligands such as Wnt5a but not “canonical” Wnt ligands such as Wnt3a.^27^,^28^ Like canonical Wnts, PCP Wnts act through frizzled (Frzld) coreceptors to recruit the intracellular coupling protein disheveled but do so independently of low-density lipoprotein receptor-related protein (LRP) receptors.^28^ Instead, they recruit van Gough-like (Vangl) proteins to Frzld at the cell membrane.^29^ The outcomes of PCP signaling are stimulus- and cell-type-dependent, including microtubule organization related to focal adhesions,^30^ polarization of division along a Wnt gradient,^31^ activation of c-Jun N-terminal kinase (JNK) signaling,^32^ and activation of the cytoskeletal regulator Rho-associated coiled coil-containing kinase (ROCK).^33^,^34^

ROCK, a component of the PCP pathway, regulates F-actin reorganization after mechanical stimulation in osteoblasts,^35^–^37^ which are highly polarized cells (disruption of their polarization is associated with less ordered bone microarchitecture).^38^ Filamentous actin forms an organized network that, together with the microtubule cytoskeleton, is itself deformed by mechanical stimulation.^39^ In addition to regulating actin stress fibers, ROCK signaling determines the position of the centriole, which acts as a mobile microtubule organizing center^40^,^41^ and is required for G1→S progression of proliferating cells.^42^ Progression past S phase to cell division must involve disruption of both actin and tubulin components, which repolymerize in order to segregate the sister chromatids and organelles.^43^–^46^

There is other evidence that noncanonical Wnt signaling may play a role in adult bone homeostasis; a number of components of the PCP pathway continue to be expressed in adult bone, and the pathway-activating ligands Wnt5a^47^,^48^ and Wnt16^49^,^50^ influence bone mass and architecture. Frizzled receptors have previously been associated with bone geometric traits,^51^ and their intracellular coupling protein disheveled is involved in bone regeneration.^52^ Disruption of the PCP pathway, as when the core pathway component van Gogh like (Vangl)2 is mutated, as in the *loop tail* (*Lp*) mouse model, leads in utero to aberrant skeletal patterning by altering the shape of the early limb bud.^26^,^53^ In addition, homozygous *Vangl2*^*Lp/Lp*^ develop a severe neurulation defect, craniorachischisis, in which the neural tube remains almost entirely open from midbrain to lower spine, and therefore do not survive postnatally.^54^ The *Lp* allele is transmitted in a semidominant fashion, with heterozygotes being viable but displaying a characteristic kinked or looped tail. To our knowledge, the postnatal skeletal phenotype of *loop tail* mice has not yet been reported.

Here, we demonstrate for the first time to our knowledge that, in addition to its well-established roles during skeletal development, PCP signaling also plays a role in the adult skeleton by orienting the direction of osteoblast division in response to dynamic strain. In this way, it contributes to the mechanisms whereby bone cells are able to produce and adapt bone architecture to be structurally appropriate for customary load bearing.

## Materials and Methods

### Cell culture and treatment

GSK 269962 (GSK), a selective inhibitor of purified human ROCK1 and ROCK2,^55^ and carrier-free recombinant human sclerostin (rhSOST) protein were from Tocris (Bristol, UK) and dissolved in ethanol or PBS, respectively. rhSOST pretreatment was always 1 hour before strain, whereas GSK was added either before or after strain as indicated in the text. 17β-estradiol (E2) was from Sigma-Aldrich (Dorset, UK) and dissolved in molecular-grade ethanol. Cells were maintained in phenol red-free DMEM containing 10% heat-inactivated fetal calf serum (FCS; PAA, Somerset, UK), 2 mM L-glutamine, 100 IU/mL penicillin, and 100 IU/mL streptomycin (Invitrogen, Paisley, UK) (complete medium) in a 37°C incubator at 5% CO_2_, 95% humidity as previously described.^13^,^56^

Primary cortical long bone–derived mouse osteoblasts were explanted from young adult 17- to 24-week-old mice as previously described^13^,^57^,^58^ and always used at passage 1. These cells have been extensively characterized by our group and are responsive to physiological mechanical strain.^57^,^58^ For strain experiments, cells were seeded at an initial density of 10,000 cells/cm^2^ on custom-made plastic slides and allowed to settle overnight before being flooded in 5 mL/slide of complete medium. After 24 hours, cells were serum depleted in 2% charcoal-dextran stripped FCS overnight before exposure to strain or treatment.

Saos-2 cells were as previously described.^13^ We and others have previously established that these cells consistently express markers of osteoblastic differentiation, including osteocalcin and, in a confluent state, sclerostin.^13^,^56^,^59^ In response to strain, the expression of numerous “mechanosensory” genes is altered, and they increase their rate of proliferation after strain or treatment with estradiol similarly to cortical long bone–derived primary osteoblasts.^13^,^56^

28/I2 chondrocytes, NIH-3T3 fibroblasts, and PC-3 prostatic carcinoma cells were kindly gifted by Dr A Muhkerjee (Royal Veterinary College, London, UK), Dr C Whiting (University of Bristol, Bristol, UK) and Prof D Bates (University of Bristol), respectively.

### Straining cells in vitro

For all experiments, cells were cultured on custom-made plastic strips, and strain was applied essentially as previously described^58^ through a brief period of 600 cycles of four-point bending of the strips with a peak strain of 3400 µε (unless otherwise stated) on a Zwick/Roëll materials testing machine (Zwick Testing Machines Ltd., Leominster, UK) with strain rates on and off of ∼24,000 µε/s, dwell times on and off of 0.7 seconds, and a frequency of 0.6 Hz.

### Indirect immunodetection

Anti-human and mouse Ki-67 and anti-paxillin primary antibodies were all from Santa Cruz Biotechnology (Heidelberg, Germany). Anti-PCM1 was from New England Biolabs (Herts, UK). NL557 (red) conjugated donkey secondary antibodies were from R&D Bioscience (Bristol, UK). Alexa 488 goat anti-rabbit and rhodamine-conjugated phalloidin were from Life Technologies (Paisley, UK). Fluoroshield aqueous mountant containing DAPI nuclear counterstain was from Sigma-Aldrich.

For anti-mouse Ki-67 staining, the antigen was retrieved by heating in PBS with 0.5% v/v Triton X-100 (Sigma, Poole, UK), blocked in 1% bovine serum albumin (BSA) solution for 30 minutes, 10% rabbit serum for 1 hour, and then 10% horse serum for 1 hour at room temperature. For human Ki-67 staining, a 1-hour block in horse serum was sufficient. In both cases, the primary antibody was used at a 1:100 dilution overnight at 4°C. Other antigens were detected by first permeabilizing cells in 0.5% v/v Triton X-100, blocked for 1 hour in 10% horse serum, and incubated with the appropriate primary antibody at a 1:200 dilution overnight. The next day, cells were washed 3 × 5 minutes in PBS with 0.5% v/v Triton X-100 before being incubated with a 1:200 dilution of the secondary antibody for 1 hour at room temperature in the dark. Cells were then washed again 3 × 5 minutes and mounted in Fluoroshield. Images were captured on a Leica DMRB microscope with an Olympus DP7.2 digital camera, which was carefully aligned using an Osteomeasure histomorphometry suite (Osteometrics, Decatur, GA, USA) such that the long axis of the field of view matched the axis of strain.

### Determining the orientation of division

Ki-67 staining is brightest during mitosis. It is, therefore, possible to image Ki-67-stained mouse osteoblasts, Saos-2, NIH-3T3, 28/I2, or PC-3 cells at low power (10 ×) such that mitotic cells are predominantly visible. These are readily identifiable whenever two daughter cells with a typical rounded morphology are observed in close proximity to each other. Sequential images were taken along the whole stained length of the slide, requiring approximately 40 fields typically providing ∼50 to 80 suitable mitotic nuclei per slide. These images were then exported to Image J (NIH, v1.46), where the in-built angle analysis tool was used to draw a line through the center of each pair of nuclei. Cells in late anaphase or telophase were analyzed because distinct daughter nuclei are visible in these stages but not while the sister chromatids are aggregated at the metaphase plate. The horizontal axis (90^o^) represents the direction in which the slide was bent and is, therefore, the direction of strain in tension; a perpendicular plane in the z direction through the cell bodies would be expected to experience unquantifiable compression. Analyses presented in this article represent 682 ± 40 mitotic nuclei per group. This number was not predetermined but represents all observed dividing nuclei in 3 to 5 independent experiments or cells from independent mice.

**Figure 1:**
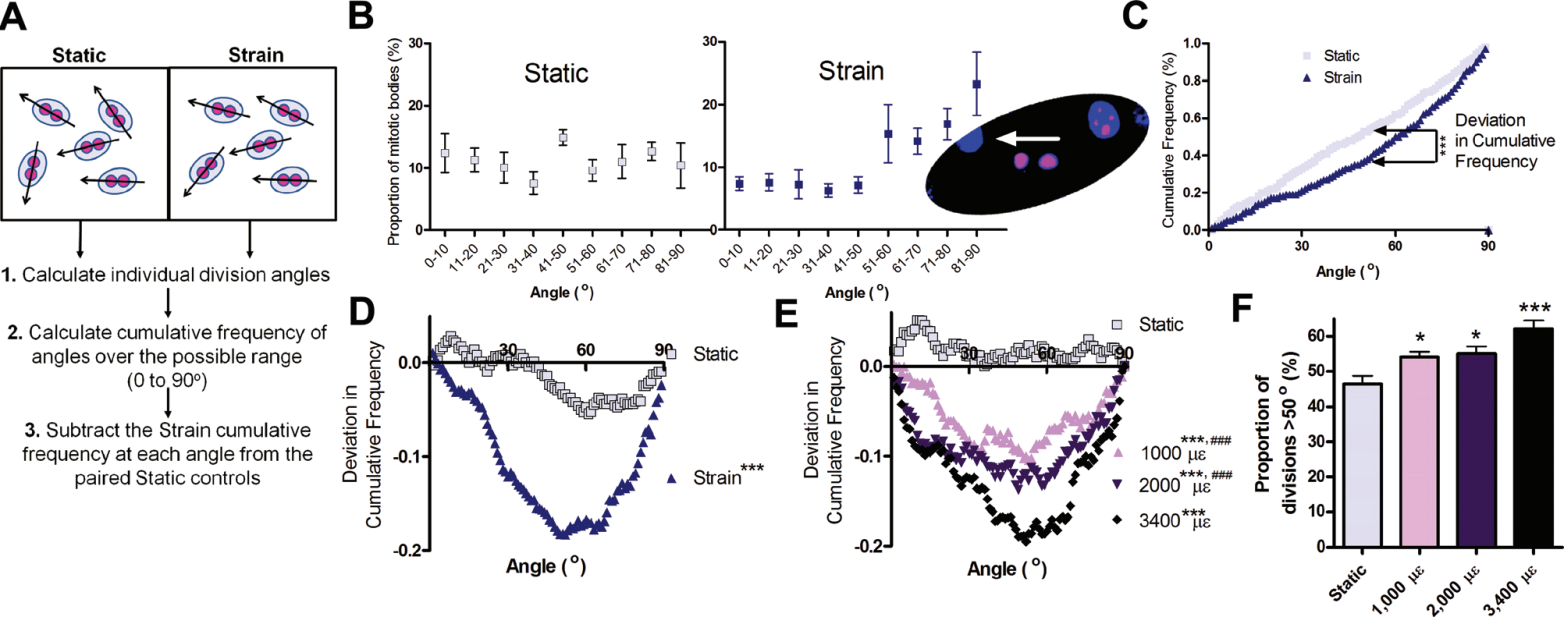
Strain orients divisions of osteoblast-like cells. (*A*) Schematic representation of the process used to analyze the orientation of divisions after strain. (*B*) The proportion of observed nuclear segregation angles between pairs of daughter nuclei, recorded within 10^o^ brackets in static or strained cultures of primary mouse osteoblasts 24 hours after stimulation. Ninety degrees is parallel, whereas 0^o^ is perpendicular to strain. Insert: Ki-67-stained (red) mitotic cell with DAPI (blue) nuclear counterstain; the arrow shows the direction of division. Reanalysis of (*B*) to illustrate in (*C*) the cumulative frequency of divisions and in (*D*) derivation of the cumulative frequency deviation analysis comparing mitotic directionality in static cultures relative to random (*x* axis) and in strained cultures relative to static controls. These studies were undertaken in Saos-2 cells 24 hours after being subjected to 3,400 µε. (*E*) Cumulative frequency deviation analysis for Saos-2 cells 24 hours after being subjected to the indicated peak strain (µε) magnitudes. (*F*) Quantification of the proportion of Saos-2 divisions occurring at angles greater than 50^o^ 24 hours after being subjected to the indicated peak strain (µε) magnitudes, *n* = 12. *p < 0.05, ***p < 0.001 versus static controls, ^###^*p* < 0.001 versus 3400 µε.

The simplest way to analyze the direction of division is to determine the proportion of observed divisions occurring within angle ranges. The methodology employed is illustrated in Fig. 1*A* and an example of this analysis is provided in Fig. 1*B* to provide an intuitive representation of the data. However, comparing experimental treatments using this proportion analysis requires artificial groupings of observed divisions in angle ranges and complicates statistical analysis unless an arbitrary fixed relationship between proportion and angle range is assumed.

To analyze the direction of cell polarity in other contexts, particularly during development, previous articles have compared the frequency of observed divisions at angles radiating from a reference direction.^60^–^62^ This cumulative frequency analysis approach was adopted in the present study. For clarity of presentation, the deviation in cumulative frequency over the range of possible angles was subsequently used. To obtain this, the cumulative frequency distribution of divisions in static control slides is compared with the expected situation if cell division were completely random, thus providing a negative control in each experiment. Cumulative frequencies on strained slides are compared with their respective static controls. Thus, the deviation in cumulative frequency (DevCF) of a strained slide from a static slide was calculated at each angle “n^o^” by comparing the number of observed divisions (Obs) at angles lesser than or equal to angle n^o^ as a proportion of the total number of observed divisions (Total) on strained (_S_) slides, and then subtracting the equivalent value at angle n^o^ from static control (_C_) slides not subjected to strain:


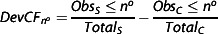


The equivalent calculation for static slides simply used the proportion of divisions expected at each angle n^o^ in a hypothetical perfectly random culture as the baseline.

Deviation in cumulative frequency analysis holds the considerable advantage that differences between groups can be analyzed by continuous ANOVA without requiring artificial groupings within angle brackets or the assumption of a fixed arithmetic relationship between direction and the proportion of divisions occurring in that direction. In addition, the point of maximum divergence, referred to in the text as the “inflection point,” indicates the angle beyond which strain preferentially orients divisions: This can be compared between treatment groups by calculating the inflection point in individual repeat experiments.

### Determining centriole orientation

To determine the position of PCM-1 staining relative to the center of each G1/S phase nucleus, a similar approach to that used to determine the orientation of division was used. After imaging of the slides at high power (40 ×), PCM-1 and Ki-67 double-labeled images were exported to Image J, where the center of each nucleus was approximated. The Image J macro presented below was used to approximate the center of each nucleus to minimize interobserver variability (blinded comparison by two independent observers) in determining the center of each nucleus relative to the punctate PCM-1 staining. This macro erodes each nucleus toward the center, thus providing a more objective measure. This was not necessary when analyzing the orientation of division, above, as each daughter nucleus was identifiable as a single, small point allowing objective quantification. (The “notes” should be removed before running the macro.)

<Note: Having opened the PCM-1, Ki-67, and DAPI images in Image J, run the macro with the DAPI image selected.>

run(“Make Binary”);

run(“Erode”); run(“Erode”); run(“Erode”); run(“Erode”); run(“Erode”); run(“Erode”); run(“Erode”); run(“Erode”); run(“Erode”); run(“Erode”);

<Note: The number of erodes will depend on the size and shape of the nuclei being analyzed.>

run(“Channels Tool… ”);

run(“Blue”);

run(“Close”);

run(“Images to Stack”, “name=Stack title=[] use”);

run(“Z Project…”, “start=1 stop=3 projection=[Max Intensity]”);

selectWindow(“Stack”);

close();

Deviation in cumulative frequency of observed centriole orientation analyses were carried out in the same way as when analyzing the direction of division. The analysis presented represents 464 ± 37 G1/S-phase nuclei. This number was not predetermined but represents all observed G1/S-phase nuclei with visible PCM-1 staining in three independent experiments.

### Scanning electron microscopy

All procedures involving mice were in accordance with the Institutional Animal Care and Home Office, UK, guidelines and approved by the ethics committee of University College London. *Vangl2*^*Lp/+*^ female mice were bred and genotyped as previously described.^63^ Tibias were disarticulated and immersed in PBS containing 10% w/v collagenase solution with 0.1% sodium azide (Sigma). Legs were incubated at 37°C for 48 to 72 hours, by which point all surrounding muscle and soft tissue had been digested. The collagenase solution was decanted, bones were washed briefly in milli-Q water, and dehydrated overnight. Bones were then gold-palladium coated and imaged on a Quanta 400FEI scanning electron microscope. Images were captured at 15 KV with a spot size of 3. Images are representative of the medial aspect of the tibial midshaft of three 5-week-old wild-type (WT) and *Vangl2*^*Lp/+*^ female mice.

### Determination of bone structure

After euthanization, legs were stored in 70% ethanol and the whole femur imaged by µCT using the SkyScan 1172 (SkyScan, Kontich, Belgium) with a voxel size of 4.8 µm (110 mm^3^). The scanning, reconstruction, and method of analysis have been previously reported.^64^ We evaluated the effect of *Vangl2* mutation on the trabecular (0.25 to 0.75 mm proximal to the distal femoral physis) and cortical site (femoral midshaft), according to ASBMR guidelines.^65^

### Statistical analysis

All bars represent the mean ± SEM. Two groups were compared by independent samples *t* test, whereas comparisons of more than two groups was by ANOVA with post hoc Bonferroni correction. Comparison of deviation in cumulative frequency plots was by continuous ANOVA against angle in GraphPad Prism (v.4.03, GraphPad Software Inc., La Jolla, CA, USA). The “inflection point” indicates strain reorients divisions that, in static cultures, would have occurred at angles smaller than the inflection point such that they occur preferentially beyond this point. This was calculated as the raw maximum deviation between the cumulative frequency of static and strain cultures in each independent repeat experiment.

## Results

### Mechanical strain reorients subsequent osteoblast divisions

Exposure of primary cultures of adult mouse-derived osteoblast-like cells in vitro to a short period of physiologically-relevant dynamic strain by four point bending of their substrate^58^ rapidly initiates proliferation, indicated by a significant increase in the proportion of cells staining positive for the proliferation marker Ki-67 (Supplemental Fig. S1*A*). This increase, evident within 1 hour, is sustained for at least 24 hours, at which point no differences are observed between the proportions of cells in different stages of the cell cycle (Supplemental Fig. S1*B*), as assessed in situ using the nuclear pattern of Ki-67 staining.^58^,^66^

In control cultures of cells not exposed to dynamic strain, the direction of segregation of Ki-67-stained anaphase/telophase nuclei was random (Fig. 1*A–C*). In cultures exposed to strain 24 hours previously, a greater proportion of these planes of division were oriented toward the principal direction in which strain had been applied. This indicates that the daughter cell nuclei were preferentially segregated parallel to the direction of strain (Fig. 1*A*, *B*). Consequently, the cumulative frequency of observed divisions over the range of possible angles (0^o^ to 90^o^) was significantly different between static and strained cultures (Fig. 1*C*). This shift was analyzed by computing the deviation in cumulative frequencies, relative to random (i.e., the *x* axis), of observed divisions over the range of possible directions in four independent repeat experiments. In unstrained cultures, the cumulative frequency of direction did not deviate significantly from random, whereas in strained cultures, this deviation was statistically significant (Fig. 1*D*). In human osteoblast-like cells (Saos-2), which also proliferate after strain (Supplemental Fig. S1*C*), the magnitude of deviation in cumulative frequency of division angles was related to peak strain magnitude (Fig. 1*E*). The proportion of divisions occurring more closely parallel to that of strain exposure, at angles greater than 50^o^, was significantly greater in strained than static cultures, and the magnitude of this effect was related to peak strain magnitude (Fig. 1*F*).

### Strain orients division of fibroblastic and prostatic carcinoma but not chondrocytic cells

**Figure 2:**
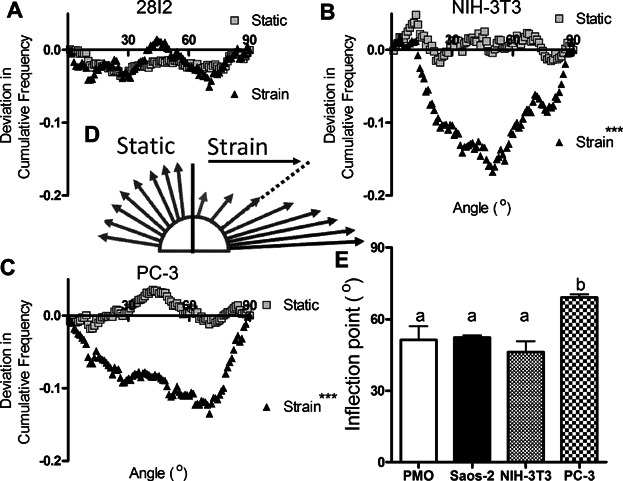
Strain orients division of fibroblastic and prostatic carcinoma but not chondrocytic cells. Cumulative frequency deviation analysis for (*A*) chondrocytic 28I2 cells, (*B*) fibroblastic NIH-3T3 cells, and (*C*) prostatic carcinoma PC-3 cells 24 hours after being subjected to strain. (*D*) Schematic representation of strain's effect on the direction of division, indicated by arrows, with the dashed line indicating the inflection point, the point beyond which strain increases the relative frequency of orientation. (*E*) Quantification of the inflection point 24 hours after strain in cultures of primary mouse osteoblasts (PMO), Saos-2 cells, NIH-3T3 cells, and PC-3 cells from four repeat experiments. *** *p* < 0.001 versus static controls. Bars with the same letter above them were not significantly different from each other (*p *> 0.05).

To test whether the effect of strain on orientation of division was specific to osteoblast-like cells, we subjected prostatic carcinoma, chondrocyte- and fibroblast-like cells to similar periods and magnitudes of strain. In human immortalized 28/I2 chondrocytes, strain had no effect on the orientation of division (Fig. 2*A*). Mouse NIH-3T3 fibroblasts and human PC-3 prostatic carcinoma cells oriented their division after strain (Fig. 2*C*, *D*). The point of maximum deviation in cumulative frequency between static and strained cultures, the “inflection point” beyond which strain preferentially orients divisions, was not significantly different between NIH-3T3, primary mouse osteoblastic cells, and Saos-2 cells, but was significantly greater in PC-3 cells (Fig. 2*D*, *E*).

### Strain-related orientation of osteoblast-like cell division involves ROCK signaling

**Figure 3:**
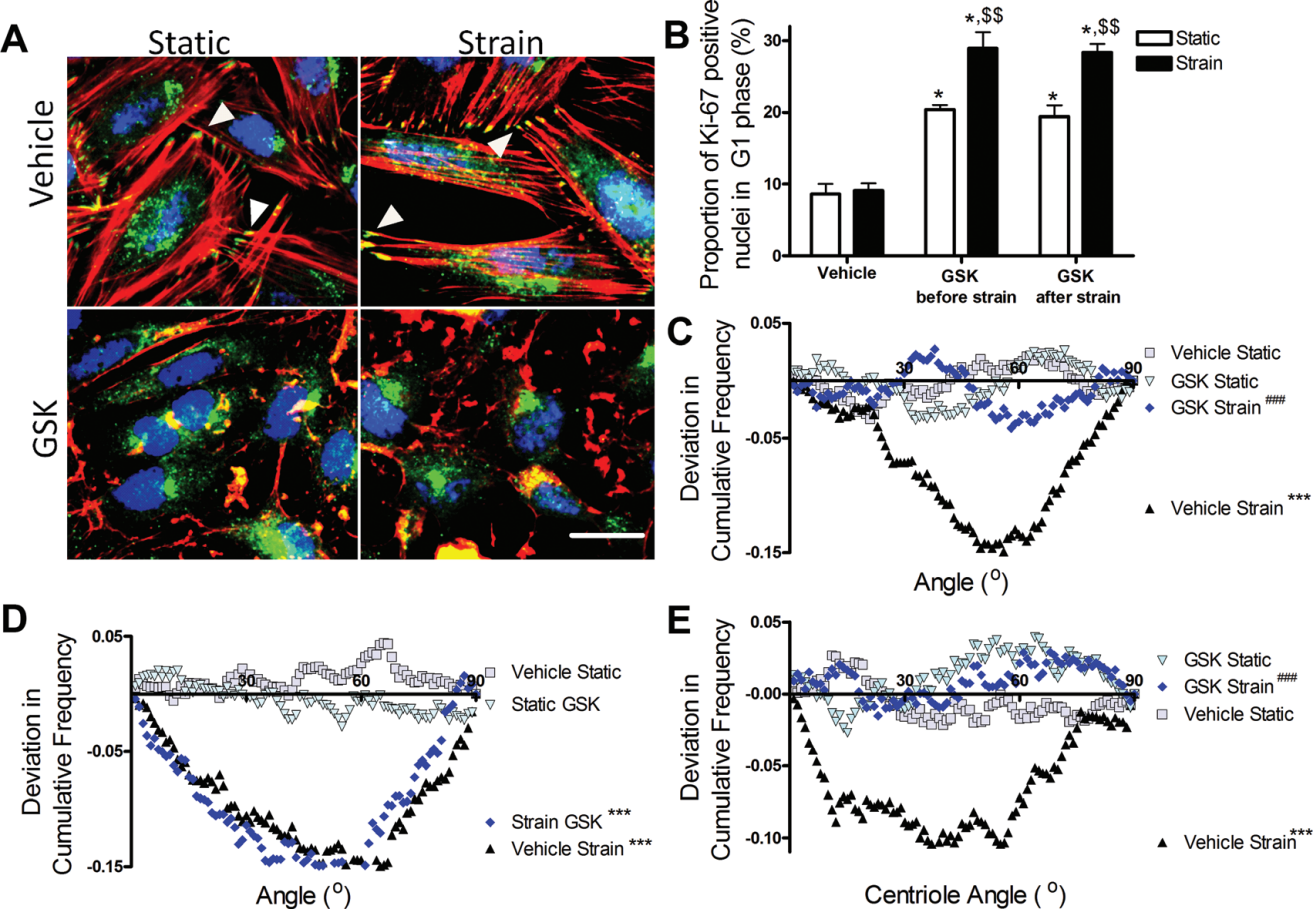
ROCK activity mediates strain-related orientation of cell divisions and centriole alignment. (*A*) Osteoblast-like cells (Saos-2) were harvested 1 hour after exposure to strain with or without 30-minute pretreatment with 1 µM GSK269962 (GSK). Representative images show F-actin (red), the focal adhesion protein paxillin (green), and the nucleus (blue). Arrows indicate paxillin colocalization with F-actin in terminal blebs likely representing focal adhesions in the vehicle-treated cells. Scale bar = 50 µm. (*B*) Saos-2 cells were treated with vehicle or with 1 µM GSK269962 1 hour before or after exposure to strain and fixed 24 hours after strain for Ki-67 in situ cell cycle analysis. The proportion of cells with a pattern of nuclear staining indicative of G1 are shown. Bars represent the mean ± SEM, *p < 0.01 versus vehicle static, $$*p* < 0.01 versus the relevant GSK-treated static group. Deviation in cumulative frequency analysis in vehicle-treated cells; (*C*) cells treated with GSK 1 hour before strain and (*D*) cells treated with GSK 1 hour after strain. (*E*) Deviation in cumulative frequency analysis of PCM-1 position relative to the center of G1/S-phase nuclei in vehicle or GSK pretreated cells 1 hour after exposure to strain. ***p < 0.001 versus respective static controls, ^###^*p* < 0.001 versus vehicle-treated and strained cells.

PCP signaling regulates the direction of cell division during zebrafish development^21^,^22^ and a number of components of this pathway are expressed in adult bone. ROCK is one component of this pathway that also orients the osteoblast cytoskeleton after strain.^35^ In our experiments, blockade of ROCK1-2 with GSK269962 1 hour before the application of strain to Saos-2 cells disorganized the actin cytoskeleton, prevented its subsequent reorganization (Fig. 3*A*), but did not cause marked disruption of the tubulin network (Supplemental Fig. S2*A*). Consistent with ROCK's role in G1→S progression, its inhibition increased the proportion of Ki-67-positive cells in the G1 phase of the cell cycle (Fig. 3*B*). Strain caused a further significant increase in the proportion of Ki-67-positive cells with a pattern of staining indicative of G1 irrespective of whether ROCK was inhibited 1 hour before or after exposure to strain (Fig. 3*B*). ROCK inhibition 1 hour before strain prevented orientation of divisions in three independent experiments (Fig. 3*C*). In contrast, ROCK inhibition 1 hour after imposition of strain did not prevent orientation of division (Fig. 3*D*).

To test whether the rapid ROCK-mediated effect of strain in orienting subsequent divisions is achieved by determining the position of the centriole,^41^ we analyzed the orientation of pericentriolar matter (PCM)-1 relative to the center of each nucleus. Double labeling with Ki-67 enabled cells in G1/S phase potentially able to replicate to be exclusively analyzed (Supplemental Fig. S3). In unstrained cultures, centriole position was random, but within 1 hour of exposure to strain, it was preferentially oriented parallel to the strain direction (Fig. 3*E*). Centriole orientation was prevented by ROCK blockade 1 hour before strain in three independent experiments (Fig. 3*E*). Other hallmarks of PCP signaling observed in osteoblast-like cells after strain include rapid upregulation of *Wnt5a* (Supplemental Fig. S4*A*), the predominant Wnt ligand expressed during osteoblast differentiation,^67^ which is able to activate both canonical and PCP pathways.^68^ Increased phosphorylation of the PCP target^33^ JNK was also observed in cells exposed to strain as previously reported by others,^69^ whereas there was no significant change in the expression of the canonical ligand *Wnt3a* at this time (Supplemental Fig. S4*B*, *C*).

### Strain independently influences the rate and orientation of osteoblast division

**Figure 4:**
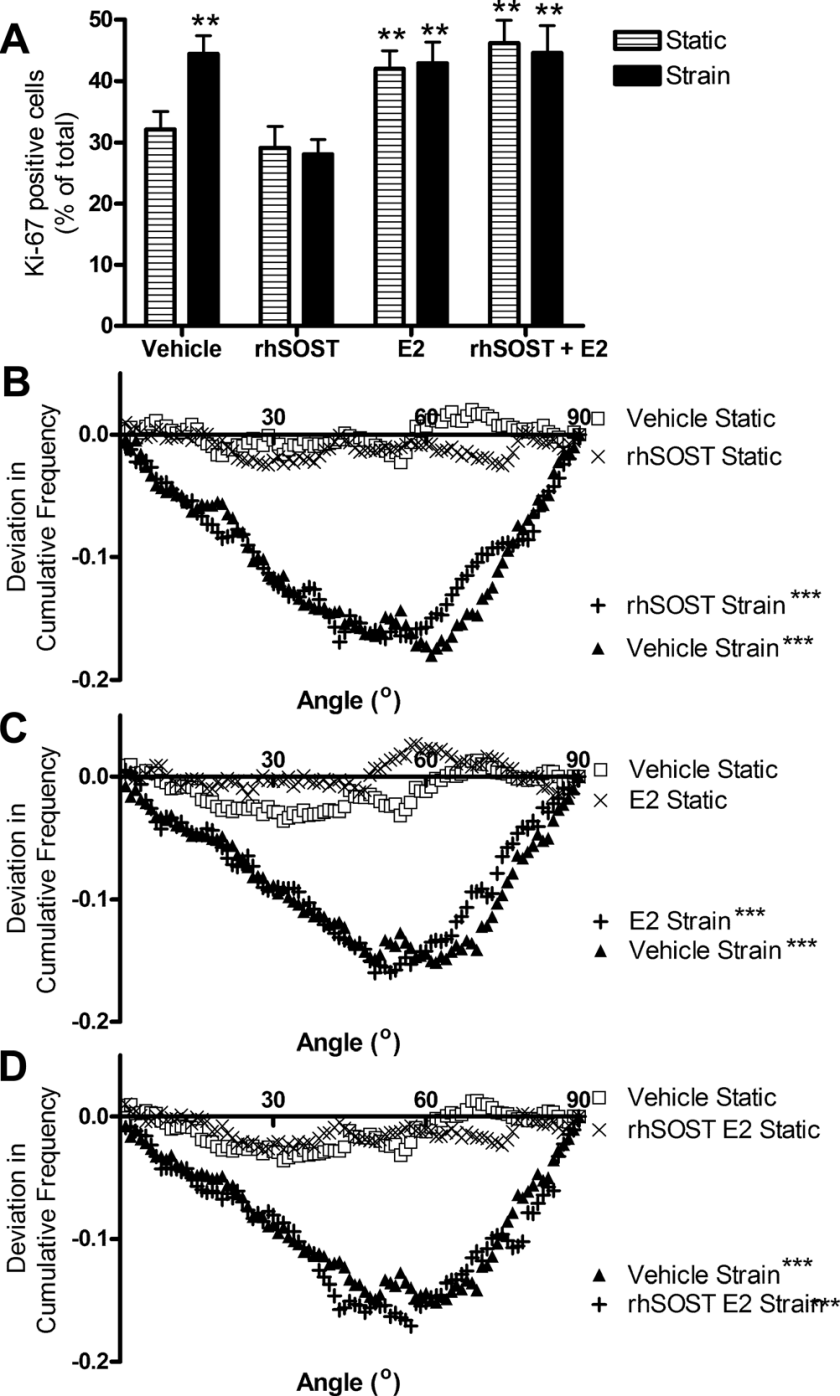
Orientation of division occurs independently of strain-induced proliferation. Saos-2 were subjected to strain with 10 ng/mL recombinant human sclerostin (rhSOST), 1 µM E2, rhSOST and E2, or vehicle pretreatment and fixed 24 hours later. (*A*) The proportion of total cells stained positive for Ki-67 was determined, *n* = 8. (*B–D*) Deviation in cumulative frequency analysis of division angles in the indicated treatment groups. (*A*) ** *p* < 0.01 compared to vehicle static. (B-D) **p < 0.01, ***p < 0.001 versus respective static controls.

The role of canonical Wnt signaling in regulation of strain-related orientation of division was examined using sclerostin, a predominantly osteocyte-derived Wnt antagonist whose expression is downregulated by strain.^16^ Sclerostin is so potent a regulator of bone formation that sclerostin neutralizing antibodies are currently in clinical trials for the treatment of osteoporosis.^70^ Pretreatment of osteoblast cultures with sclerostin prevents increased proliferation after strain (Fig. 4*A*) but does not affect strain's ability to orient cell divisions (Fig. 4*B*). As we have previously reported,^13^ treatment of Saos-2 cells with 1 µM E2 increased proliferation similarly to strain, with no additive effect when the two stimuli are combined, but sclerostin pretreatment does not prevent the increase in proliferation caused by E2 (Fig. 4*A*). Treatment with E2 before strain did not alter strain's ability to orient divisions (Fig. 4*C*), and strain oriented the divisions triggered by E2 in the presence of sclerostin (Fig. 4*D*) in three independent experiments. This finding suggests strain influences the orientation of division independently of the processes that increase the rate of division and also independently of canonical Wnt signaling.

### Vanlg2 mediates strain-related orientation of division and influences bone architecture

**Figure 5:**
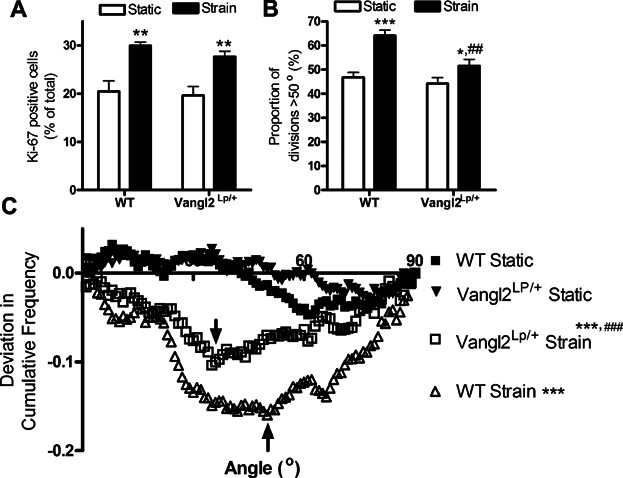
Mutation of *Vangl2* impairs orientation after strain. (*A*) Primary osteoblasts derived from WT or *Vangl2^Lp/+^* mice were subjected to strain and fixed 24 hours later. The proportion of cells stained positive for Ki-67 was determined, *n* = 8. (*B*) The proportion of divisions occurring at angles greater than 50^o^ was quantified, *n *= 4 representing 4 independent cultures. (*C*) Deviation in cumulative frequency analysis of division angles in cultures from WT or *Vangl2^Lp/+^* mice. Arrows approximately indicate the inflection point. **p < 0.01, ***p < 0.001 versus respective static controls, ^##^*p* < 0.01, ^###^*p* < 0.001 versus strained cultures from WT mice.

Sclerostin's mode of action is to antagonize Wnt by binding the LRP5/6 component of osteoblasts' LRP5/6/Frzld receptor.^71^ It thus has no direct effect on LRP-independent noncanonical Wnt/PCP signaling. To investigate the potential roles of PCP signaling, primary osteoblasts were derived from the long bones of *loop-tail* (*Lp*) mice^63^ that have a mutation affecting the core PCP component Vangl2. Heterozygous (*Vangl2*^*Lp/+*^) mice were used because the *Vangl2*^*Lp/Lp*^ homozygote is lethal and exhibits defective skeletal patterning.^53^ Osteoblast proliferation in response to strain was not affected by the mutation of a copy of *Vangl2* (Fig. 5*A*). However, a significantly smaller proportion of osteoblasts from *Vangl2*^*Lp/+*^ mice oriented their division in response to strain than those derived from WT mice (Fig. 5*B*, *C*). In addition, the “inflection point” of cultures from *Vangl2*^*Lp/+*^ mice was smaller than for WT (Supplemental Fig. S5*A*, *B*). Thus, strain orients division of fewer cells over a smaller arc of influence in cultures from *Vangl2*^*Lp/+*^ mice than WT.

**Figure 6:**
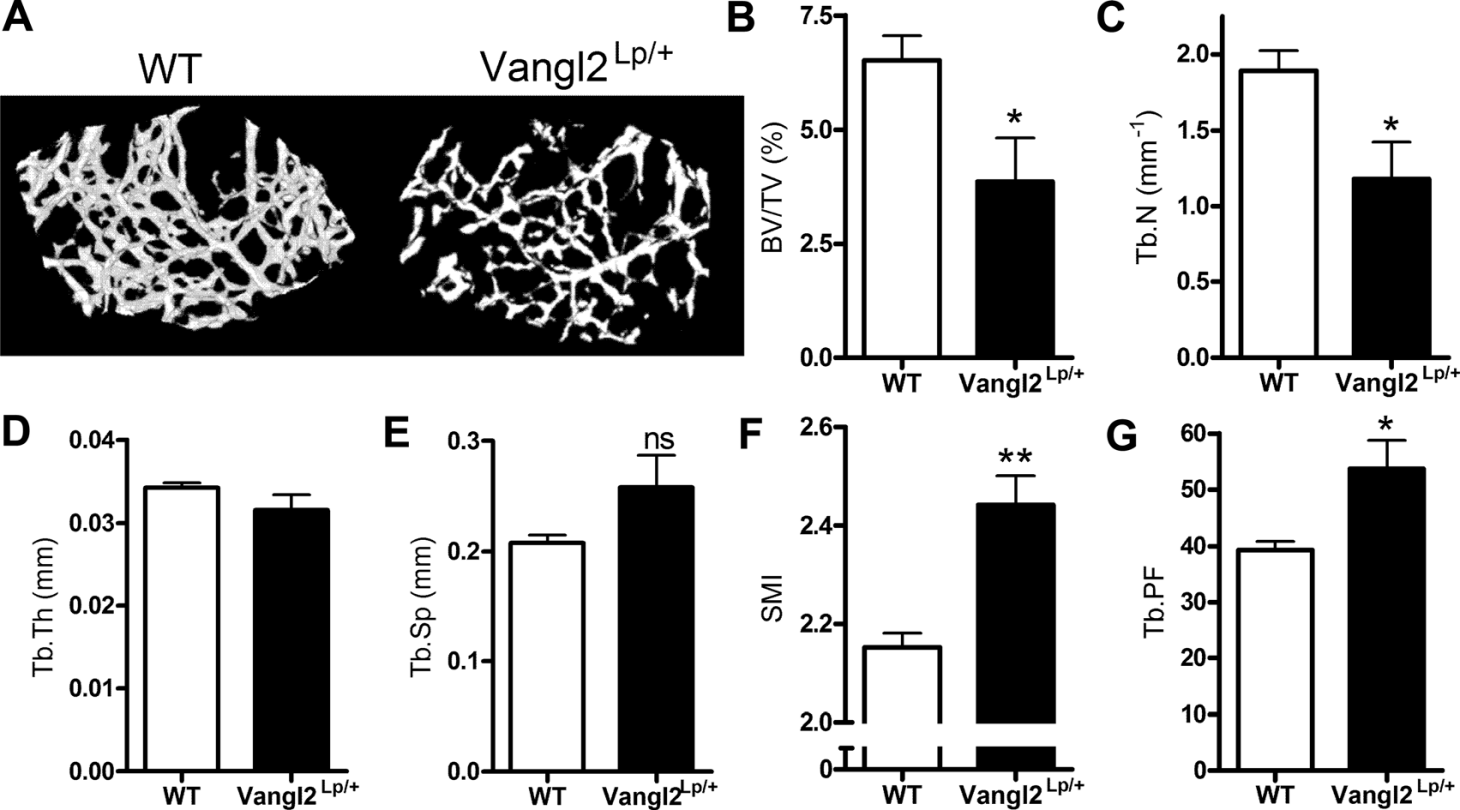
Mutation of *Vangl2* alters postnatal trabecular bone structure. (*A*) Representative 3D µCT reconstructions of distal femur trabecular bone of 5-week-old female WT or Vangl2Lp/+ mice. Quantification of distal femur: (*B*) trabecular bone volume fraction (BV/TV), (*C*) number (Tb.N), (*D*) thickness (Tb.Th), (*E*) separation (Tb.Sp), (*F*) structure model index (SMI), and (*G*) trabecular pattern factor (Tb.PF) of WT or Vangl2Lp/+ mice, *n* = 6. Bars represent means ± SEM. *p < 0.05, **p < 0.01 versus WT.

To determine whether PCP signaling through Vangl2 contributes to postnatal bone structure, standard architectural measures were assessed in 5-week-old female WT and *Vangl2*^*Lp/+*^ mice by µCT. Body weight, femoral lengths, and bone mineral density were not significantly different between the genotypes at this age (Supplemental Table S1), but the *Vangl2*^*Lp/+*^ mice had lower bone mass than WT rather than the high bone mass phenotype that would be expected if canonical Wnt signaling had increased to compensate for deficient PCP signaling. Trabecular bone volume fraction and trabecular number, but not trabecular thickness or separation, in the distal femur of *Vangl2*^*Lp/+*^ mice was significantly lower than in WT controls (Fig. 6*A–E*). Trabecular pattern factor and structure model index in the distal femur were significantly greater in *Vangl2*^*Lp/+*^ than WT mice (Fig. 6*F*, *G*), indicating deviation from trabecular plates toward rods, a process that occurs in humans with aging.^72^

**Figure 7:**
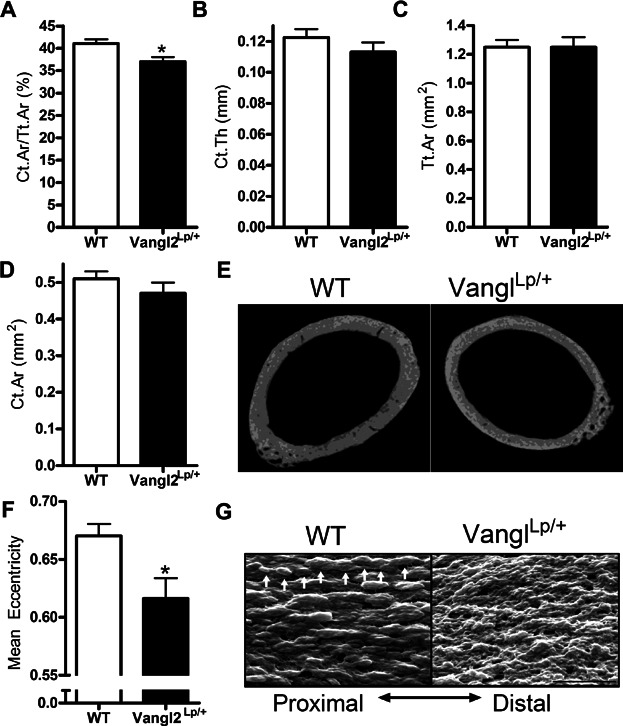
Mutation of *Vangl2* alters postnatal cortical bone structure. Quantification of mid-femoral (*A*) cortical area fraction (Ct.Ar/Tt.Ar), (*B*) cross-sectional thickness (Ct.Th), (*C*) medullary area (Ma.Ar), and (*D*) cortical bone area (Ct.Ar) of 5-week-old female WT and *Vangl2^Lp/+^* mice, *n* = 6. (*E*) Representative cross-sectional images of the mid-femoral cortical bone of WT and *Vangl2^Lp/+^* mice. (*F*) Mean eccentricity quantification in the femoral midshaft. (*G*) Representative scanning electron micrographs of the periosteal mineralizing front on the medial midshaft of collagenase-digested tibias of WT and *Vangl2^Lp/+^* littermates; scale bar = 5 µm; arrows indicate mineral bundles. Bars represent means ± SEM. *p < 0.05 versus WT.

Cortical area fraction was also significantly lower in *Vangl2*^*Lp/+*^ than in WT mice (Fig. 7*A*). No significant differences were detected in cortical cross-sectional thickness, total tissue area, cortical bone area (Fig. 7*B–D*), or medullary area (not shown) between the two genotypes. Rather, *Vangl2*^*Lp/+*^ mice had significantly altered cortical shape as quantified by eccentricity. Femoral midshaft eccentricity was lower in *Vangl2*^*Lp/+*^ than in WT mice (Fig. 7*E*, *F*) possibly indicating failure to achieve the more elliptical shape formed as a natural response to load bearing^73^ found in WT littermates. The microarchitectural basis of these changes in structure was explored by scanning electron microscopy of the medial midtibial periosteal mineralizing front. This clearly demonstrated organized crystal alignment in WT mice that was not evident in *Vangl2*^*Lp/+*^ littermates (Fig. 7*G*). Images shown are representative of three pairs of WT and Vangl2^Lp/+^ littermates from three different litters.

## Discussion

**Figure 8:**
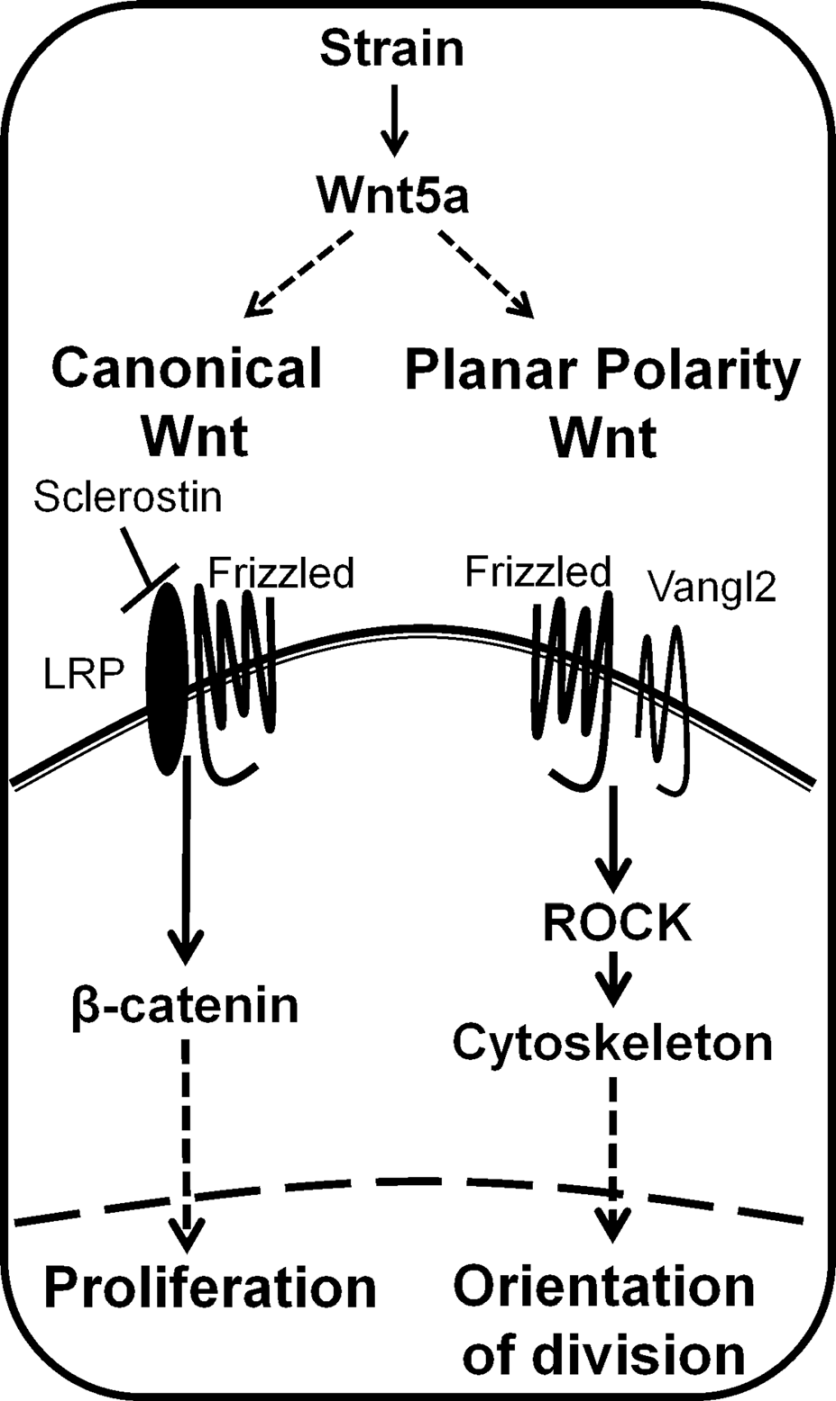
Schematic representation of the proposed mechanisms by which strain increases the rate of division and independently informs the orientation of division. Strain causes rapid upregulation of Wnt5a expression, which has the potential to act through frizzled (Frzld) in an LRP-dependent or -independent manner.^68^ Blockade of LRP-dependent canonical Wnt signaling (which may involve Wnts other than Wnt5a) with sclerostin prevents proliferation, whereas Vangl2 influences the reorientation of division but not proliferation after strain.

The data presented here confirm that brief exposure of osteoblasts to dynamic mechanical strain of physiological magnitude stimulates an increase in their rate of division. They also establish that this mechanism involves the canonical Wnt pathway. The novel contribution these data make is that they also establish that such brief exposure to dynamic strain stimulates a concurrent, yet independent, planar cell polarity–related orientation of this division (Fig. 8). This orientation of division involves ROCK-mediated centriole realignment and occurs within the first hour after strain.

As expected, ROCK inhibition disrupted the F-actin cytoskeleton and Ki-67 staining demonstrated an increase in the proportion of cells in the G1 phase of the cell cycle. Given the dramatic cytoskeletal disruption, we cannot exclude the possibility that this may have induced secondary changes in related signaling cascades that could have influenced our results; e.g., redistribution of paxillin away from structured focal adhesions. However, ROCK inhibition with GSK did not cause the same deregulation of the tubulin network, itself a load-bearing structure.^39^ Furthermore, not all cellular responses to strain were abrogated by ROCK blockade as strain increased the proportion of cells in the G1 phase of the cell cycle irrespective of whether GSK was added before or after strain. These findings are consistent with the well-recognized activation of Rho/ROCK signaling by mechanical stimulation^37^,^74^ and ROCK's involvement in the directional alignment of the osteoblastic actin cytoskeleton.^35^

The reliance on Saos-2 human osteoblastic sarcoma cells for these studies is a potential limitation. However, it was not feasible to use primary cultures for all of our studies. We and others have previously extensively characterized Saos-2 cells' osteoblastic nature and physiological responses to strain,^13^,^56^,^59^ and a very recent publication has described their use as a model to study osteoblast to osteocyte transition.^75^ Furthermore, we demonstrate here that exposure to strain orients a similar proportion of divisions over a similar arc of influence in Saos-2 cells compared with primary cortical long bone–derived mouse osteoblasts. It is interesting that strain-related orientation of division is also observed in fibroblasts that are also directionally loaded in vivo and align themselves with the loading direction, but does not occur in chondrocyte-like cells, which in vivo are also subjected to loading by hydrostatic compression.^76^

Strain-related orientation of division is not confined to primarily load-bearing tissues because in prostatic carcinoma cells, which originate from a cell type only known to be exposed to strain during glandular contraction, orientation of division also occurs. In this case, it appears to occur over a greater arc of influence than in osteoblastic or fibroblastic cells. The extent to which the initial arc of orientation determines the final orientation of the cells in each situation, and the advantage conferred, remains to be established.

It is unclear from our experiments the extent of the mechanical advantage presumed to result from this orientation of division. It is relevant in this context that *Vangl*^*Lp/+*^ mice orient their divisions over a smaller arc of influence than similarly derived cells from WT mice. Similar rates of basal proliferation and an equivalent increase in proliferation after strain suggests that osteoblastic cultures from WT and Vangl^Lp/+^ mice are comparable, although further studies are required to confirm this. Osteoblast-specific Vangl2 loop tail mutation models are not currently available, and knockdown or knockout approaches do not fully mimic the effects of this mutation as the loop tail mutation produces a dominant negative allele of Vangl2, which abrogates PCP signaling.^77^,^78^

The role of Vangl2 in the orientation of division after strain is consistent with its regulation of mitotic spindle orientation in other cell types and contexts.^79^ The characteristic looped tail of Vangl2^Lp/+^ mice is believed to develop because of defective neural tube closure, although heterozygous mice grow to adulthood and are fertile.^63^,^80^ This may be in part due to compensation for loss of Vangl2 function by Vangl1 in other tissue types. Vangl1 is a highly conserved, structurally similar paralogue of Vangl2, and is the only other known mammalian orthologue of Drosophila strabismus/Van Gogh. Both Vangl1 and Vangl2 proteins interact physically with mammalian Dvl.^81^ Moreover, Vangl1 interacts genetically with Vangl2 during neurulation.^82^ However, to our knowledge, Vangl1 has not been reported to influence skeletal patterning as Vangl2 does.^53^ It is, therefore, possible that Vangl2 is more important in bone than other tissues.

In the present study, we used young, 5-week-old recently weaned mice for several reasons: 1) to study mineralizing front morphology at a point of active growth; 2) to minimize the potential for the remaining normal Vangl2 allele in mutant mice to compensate for the deficiency of one mutated allele over the mouse's lifetime; and 3) to avoid needing to account for the confounding effects of differences in body weight altering load bearing (adult *Vangl2*^*Lp/+*^ mice are lighter than WT littermates potentially indicating systemic deficiencies).^83^,^84^ At this age, *Vangl2*^*Lp/+*^ mice have a periosteal mineralizing front on the dorso-medial surface of the tibial midshaft, which is less organized and directional than in WT mice. This specific phenotype has, to our knowledge, not previously been reported in other transgenic mouse models because scanning electron microscopy is rarely employed to characterize bone phenotypes of transgenic mice. *Vangl2*^*Lp/+*^ mice also have altered trabecular and cortical bone (micro)architecture, demonstrating a role for the PCP/Vangl2 pathway in skeletal adaptation beyond its previously reported role during in utero patterning.^24^,^53^,^85^ Given that in utero skeletal morphogenesis is also influenced by mechanical stimulation through muscle contraction,^86^ it is possible that Vangl2 facilitates functional adaptation to strain even in the prenatal period.

The search to reveal the pathway, or pathways, involved in functional adaptation in bone has been long and frustrating because, although much has been discovered, so much more needs to be revealed before a clear picture of the overall mechanisms can be made. It is clear that the most likely candidate cells for the early responses to strain are those osteocytes and osteoblasts exposed to the mechanical circumstances of the load-bearing bone tissue.^87^ The data presented here confirm that osteoblasts as well as osteocytes respond to strain in their local environment. Although surface osteoblasts may receive information regarding the strain-related circumstances of osteocytes, the data presented here support the concept that they are also capable of responding to their own local strain environment both by proliferation and orientation. The independence of proliferation and the orientation of cell division is consistent, not only with local strain being capable of stimulating division but also with systemic stimuli for increased proliferation such as estradiol being distinct from, although potentially additional to, structurally relevant architectural cues that result from local loading.^88^,^89^

In conclusion, we present data demonstrating that in addition to providing developmental cues during skeletal development,^53^ planar cell polarity signally also acts postnatally to influence orientation of osteoblast division in a manner related to these cells' immediate strain-related experience. This mechanism potentially contributes, along with other functional strain-related mechanisms, to the overall adaptive process by which bone cells respond to skeletal load bearing to ensure structurally appropriate bone architecture. It is legitimate to suppose that this effect confers some functional advantage, thus extending the range of mechanisms involved in functional adaptation in bone often described as Wolff's Law.^5^

## Disclosures

All authors state that they have no conflicts of interest.
