# Supplementary material for: Planar Cell Polarity Aligns Osteoblast Division in Response to Substrate Strain
Source: J Bone Miner Res. 2015 Feb 16;30(3):423–35. doi: 10.1002/jbmr.2377 (PMC4333081; doi:10.1002/jbmr.2377)
Supplement: Supplementary file 1 [file jbmr0030-0423-sd1.docx]

# Online supplementary materials: Supplementary table Supplementary figures 1-4 Supplementary referenceSupplementary Table 1: Quantification of body weight and standard μ-computed tomography cortical and trabecular bone parameters in 5 week old female mice. Cortical bone parameters were analysed at the femoral mid-shaft. Trabecular parameters were analysed 100 μCT lines above the growth plate. Values represent the mean ± SEM, n = 6. * p < 0.05.

|  | Wild type | *Vangl2^Lp/+^* |
| --- | --- | --- |
| Body weight (g) | 16.4 ± 0.8 | 15.1 ± 1.5 |
| Femoral length (mm) | 12.3 ± 0.2 | 12.0 ± 0.4 |
| Bone mineral density (g/cm^2^) | 0.15 ± 0.03 | 0.14 ± 0.02 |





Supplementary Figure 1: Strain increases osteoblast proliferation within 1 hour and does not alter progression through the cell cycle 24 hours later. Mouse long bone-derived osteoblasts were subjected to strain and fixed A) 1 hour or B) 24 hours later. A,B) The proportion of cells stained positive for Ki-67 was determined. A similar, significant increase in the proportion of cells stained positive for Ki-67 was observed at both time points. B) The proportion of Ki-67 positive cells in different stages of the cell cycle was also determined using the pattern of Ki-67 nuclear localisation. No significant differences were observed between the proportion of replicating cells in different stages of the cell cycle. This indicates that the divisions analysed at this time point following strain reflect mitosis of cells which entered the cell cycle following exposure to strain. C) Saos-2 cells were subjected to strain and fixed 24 hours later. The proportion of total cells stained positive for Ki-67, and the distribution of Ki-67 positive cells in different stages of the cell cycle was analysed, indicating that these cells respond to strain similarly to mouse osteoblasts. Bars represent the mean ± SEM. ** p < 0.01


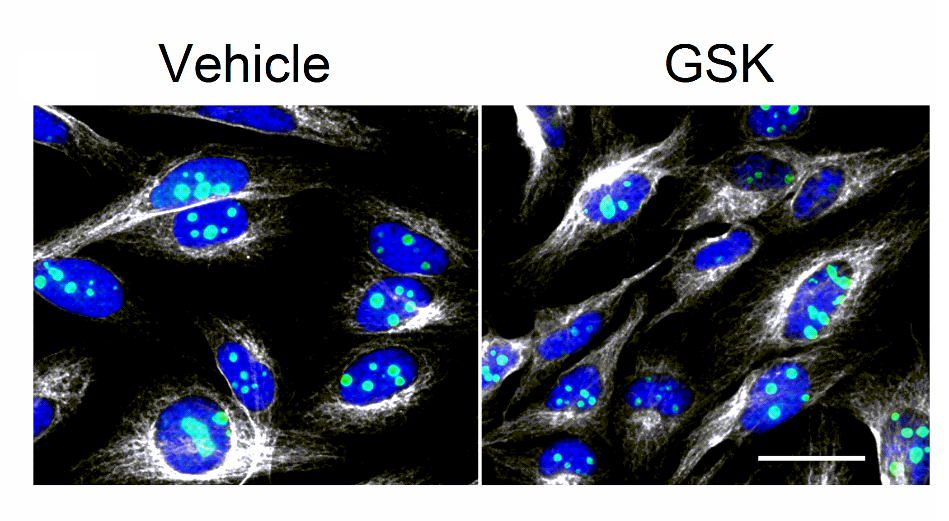


**Supplementary Figure 2:** ROCK blockade does not disrupt the tubulin network, increases the proportion of cells in G1 of the cell cycle and does not prevent cell cycle entry following strain. Saos-2 cells were treated for 1 hr with vehicle or 1 μM GSK269962 and fixed for immunofluorescence visualisation of tubulin (white) double-stained with Ki-67 (green) and nuclear DAPI counter-stain (blue). Scale bar = 125 μm.

Supplementary Figure 3 Immunofluorescent detection of the centriole protein PCM-1 and illustration of the method used to calculate centriole position relative to the centre of G1/S phase nuclei. A) Representative high magnification image of Saos-2 cells triple-labelled for PCM-1 (green, indicated by green arrows), tubulin (red) and nuclei (blue, DAPI). A mitotic cell is indicated by the purple arrow. Scale bar = 50 μm. B) Illustration of the centriole angle analysis method. PCM-1 (green) and Ki-67 (red) are double labelled on DAPI-counterstained nuclei. G1/S phase nuclei determined by the pattern of Ki-67 staining are shown by white arrows. Yellow arrows indicate the approximate relationship of PCM-1 to the centre of each G1/S phase nucleus. Independent analyses of PCM-1 orientation following strain were undertaken by GLG and LBM.

**

**

Supplementary Figure 4: Hallmarks of PCP signalling observed in following strain include Wnt5a up-regulation and increased JNK phosphorylation. A) Saos-2 cells were subjected to strain and harvested at the indicated time points. Wnt5a expression was quantified by qRT-PCR. A significant increase in Wnt5a was observed 30 mins following strain. B) Phospho- (p-) and total-JNK (both antibodies from Santa Cruz) were determined by western blotting essentially as previously described ([1](#_ENREF_1)) in lysates of Saos-2 cells harvested 30 mins following strain, observing increased p-JNK band density quantified relative to static groups in Image J, n=4. Actin is shown as a loading control. C) Wnt3a expression was quantified by qRT-PCR in cells harvested 30 mins following strain but its expression was not significantly altered by strain. Bars represent the mean ± SEM. * p<0.05, ***p<0.001 versus static controls.





Supplementary Figure 5: Hallmarks of PCP signalling observed in osteoblast-like cells following strain include the involvement of Vangl2. A) Primary mouse osteoblasts were derived from long bones of WT or *Vangl2^Lp/+^* mice, subjected to strain and fixed 24 hours later, n=4. The inflection point in the deviation in cumulative frequency of observed divisions was found to be significantly lower in cultures from *Vangl2^Lp/+^* than WT mice. This indicates that strain orients division over a smaller arc of influence in osteoblasts lacking one functional copy of *Vangl2*. The inflection point, schematically illustrated in (B), is approximately indicated in Fig. 3 c in the main text. Bars represent the mean ± SEM. * p<0.05, versus WT.

Supplementary reference:

1. Sunters A, Armstrong VJ, Zaman G, Kypta RM, Kawano Y, Lanyon LE, et al. Mechano-transduction in osteoblastic cells involves strain-regulated estrogen receptor alpha-mediated control of insulin-like growth factor (IGF) I receptor sensitivity to Ambient IGF, leading to phosphatidylinositol 3-kinase/AKT-dependent Wnt/LRP5 receptor-independent activation of beta-catenin signaling. J Biol Chem. 2010 Mar 19;285(12):8743-58.
